# Study on the Selective Laser Melting of CuSn_10_ Powder

**DOI:** 10.3390/ma11040614

**Published:** 2018-04-17

**Authors:** Chengyang Deng, Jinwu Kang, Tao Feng, Yunlong Feng, Xiang Wang, Pengyue Wu

**Affiliations:** 1 Key Laboratory for Advanced Materials Processing Technology, Ministry of Education, School of Materials Science and Engineering, Tsinghua University, Beijing 100084, China; an123yi@126.com (C.D.); wangxian17@mails.tsinghua.edu.cn (X.W.); 2 Beijing e-Plus 3D Tech. Co. Ltd., Beijing 100084, China; fengtao@eplus3d.com (T.F.); dengcy09@163.com (Y.F.); wugenlin@126.com (P.W.)

**Keywords:** selective laser melting, CuSn_10_ powder, laser energy density, microstructure, mechanical properties

## Abstract

The selective laser melting of tin bronze (CuSn_10_) powder was performed with a laser energy density intensity level at 210, 220, and 230 J/mm^2^. The composition was homogeneous with almost all tin dissolved into the matrix. The grain size of the obtained alpha copper phase was around 5 μm. The best properties were achieved at 220 J/mm^2^ laser energy density with a density of 8.82 g/cm^3^, hardness of 78.2 HRB (Rockwell Hardness measured on the B scale), yield strength of 399 MPa, tensile strength of 490 MPa, and an elongation that reached 19%. “Balling effect” appeared and resulted into a decrease of properties when the laser energy density increased to 230 J/mm^2^.

## 1. Introduction

Tin bronze (CuSn_10_) is widely used as bearing materials for its good mechanical properties [1]. Nowadays, mechanical alloying (MA) [2], powders metallurgy (PM) [3], and traditional casting [4] are the common methods for sintering CuSn_10_ products. The porosity and strength are the most important problems in the selection of these sintering processes. Many effective methods, such as using high quality sintered powder, reducing the oxygen content, and adding reinforcing phases, have been carried out to improve its mechanical properties [5,6]. The strength and density of the sintering specimens were improved with the usage of atomized powder and a dense sintering graphic mold [1,2,3,4,5,6]. The content of inclusions was reduced by sintering the powder under a vacuum atmosphere, as the metal particles were susceptible to reaction with oxygen at high temperatures [7]. Meanwhile, graphite could be used as a reinforcing phase, because high graphite content can improve the yield of the tin bronze powder and prevent excessive cold welding during the ductile-brittle milling process of the specimen [2].

Selective laser melting, as a new forming method, was introduced into sintering metal powder because it can selectively fuse metal powder particles using a computer-controlled laser beam. A wide range of materials can be sintered in this way with a high energy laser [8,9,10]. The properties can be improved by adjusting the process parameters.

The high thermal conductivity and reflectivity of copper alloys formed by laser melting resulted in significant heat loss and inadequate melting of the powder [11]. Recent studies pointed out the relationships between the laser energy density and the three-dimensional (3D)-printed metal properties. Wang et al. [12] explored the relations between laser energy density and the densification behavior of 3D-printed AlSi_10_Mg alloys, and the results showed that laser energy density has a significant effect on the forming of defects which could lead to poor mechanical properties of the as-printed parts. Mao et al. [13] studied the statistical influences of process parameters, and their results revealed that laser power has the strongest effect on the relative density and Vickers hardness of 3D-printed Cu-4Sn parts. Zhang et al. [14] indicated that a laser energy density over 340 J/mm^3^ can result in dense 3D-printed parts of wrought Al-Cu-Mg alloys.

In this work, additive manufacturing of pure tin bronze (CuSn_10_) powder was performed at a low laser power, and the relationship between the metal properties and laser energy density was revealed.

## 2. Experimental

The 99.9% purity tin bronze (CuSn_10_) powder employed in this study had an average size of 20–50 μm, with a composition of 90% Cu and 9.9% Sn. Bar samples were fabricated using the 3D printer EP-M100T, designed and manufactured by Beijing e-Plus 3D Tech. Co. Ltd., Beijing, China. The fabrication parameters for these bars were as follows: laser power P = 95 W, layer thickness h = 0.02 mm, hatch distance between adjacent laser passages d = 0.06 mm, and three laser energy density levels of E = 210, 220, and 230 J/mm^2^. The scanning speed was calculated using the formula v = P/(h × E × d) (mm/s).

**Figure 1:**
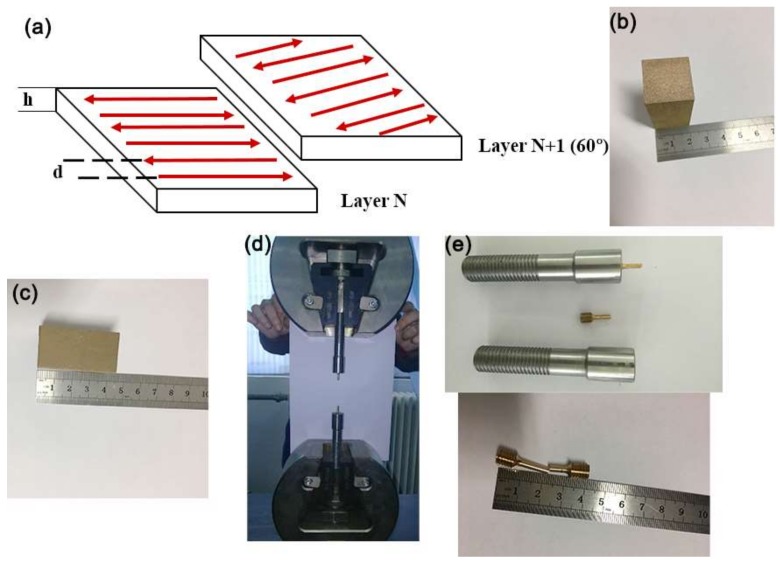
Three-dimensional (3D)-printed samples and room temperature tensile tests. (**a**) The fabrication process; (**b**) The width of three-dimensional (3D)-printed sample; (**c**) The length of three-dimensional (3D)-printed sample; (**d**) Tensile specimen machine; (**e**) The sample used for tensile test.

The fabrication process was performed under an argon atmosphere, and the content of oxygen was controlled to be below 100 ppm. The base plate was not preheated. The rotation angle of the laser scanning direction of adjacent layers was 60° (Figure 1a).

The process parameters of the hatch distance and laser energy density were varied to study their impact on properties and microstructure, as listed in Table 1.

The size of the 3D-printed bars was 20 ± 0.1 mm × 20 ± 0.1 mm × 45 ± 0.1 mm (Figure 1b,c). To ensure the consistency of the part location, samples of each experimental condition were printed one at a time, with three repetitions.

The density of the formed tin bronze specimens was determined by the Archimedes method. Hardness was measured by an HR-150A Sclerometer.

Square samples (10 mm × 10 mm × 8 mm) were made from 3D-printed bars using wire cutting. Their microstructures and surface morphologies were characterized by a Keyence VHX-6000 digital microscope (Keyence, Osaka, Japan). A JEOL JSM 6301F spectroscope electron microscope (SEM) (Zeiss, Hallbergmoos, Germany) was used to examine chemical compositions. 

In accordance with the Chinese room temperature tensile tests standard GB/T228.1-2010, mechanical properties were investigated using standard samples with a diameter of 5 mm made from the 3D-printed bars using wire cutting at the WDW-300 testing facility (Xinshijinshiyanji Tech. Co. Ltd., Jinan, China). The tensile test is shown in Figure 1d,e.

## 3. Results and Discussion

**Figure 2:**
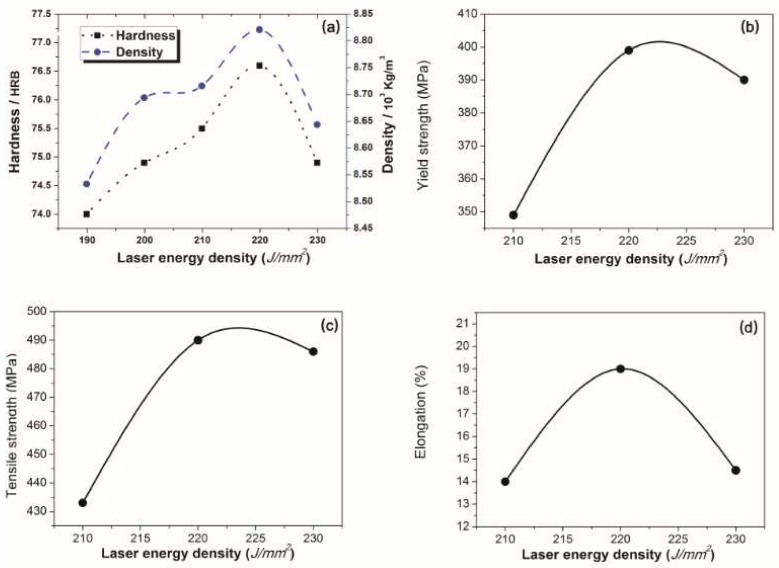
Mechanical properties at different laser energy densities. (**a**) The hardness and density of the samples; (**b**) The yield strength of the samples; (**c**) The tensile strength of the samples; (**d**) The elongation of the samples.

Figure 2a shows that the density and the hardness are in proportion to the laser energy density below the threshold value of 220 J/mm^2^, which indicates that the best properties are achieved with a laser energy density of 220 J/mm^2^. The mechanical properties of samples fabricated under 210 J/mm^2^, 220 J/mm^2^, and 230 J/mm^2^ laser energy densities were characterized by room temperature tensile tests, considering that the properties are poor when the laser energy density is less than 210 J/mm^2^. The results are listed in Table 2.

**Figure 3:**
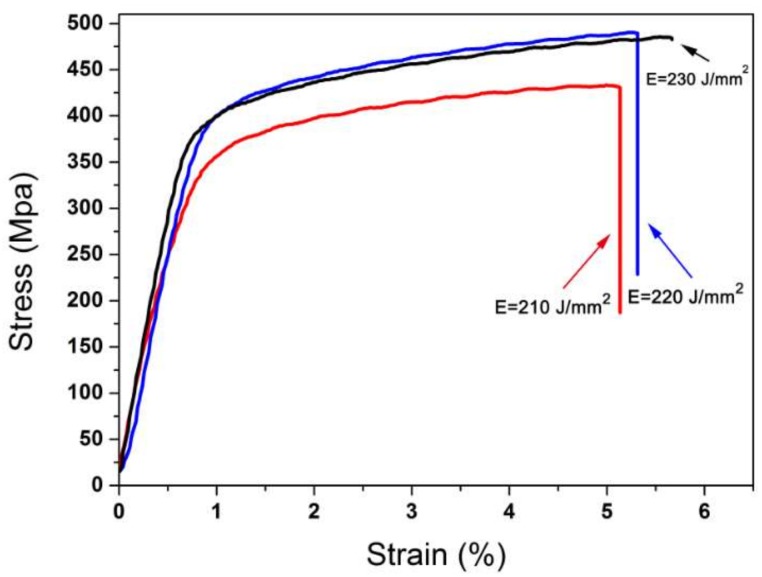
Tensile stress-strain cures of samples in different laser energy densities.

Referred from Figure 2b–d and Figure 3, the maximum density reaches 8.9 g/cm^3^, and the maximum hardness simultaneously reaches 76.6 HRB. The maximum yield strength and tensile strength are 399 MPa, 490 MPa, respectively, with a maximum elongation of 19%. Compared with the Chinese Industrial Standards YS/T 545-2006 [15], the properties are far higher than those of products made by the continuous casting process. 

**Figure 4:**
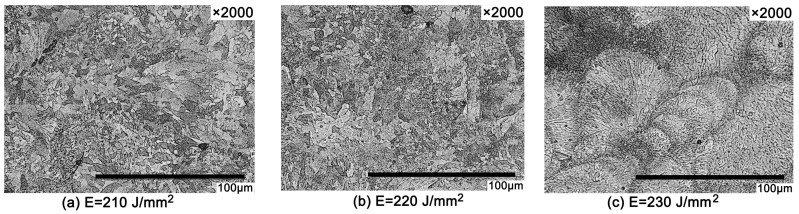
Metallographic morphology at different laser energy densities. (**a**) Metallographic morphology at 210 J/mm^2^ laser energy density; (**b**) Metallographic morphology at 220 J/mm^2^ laser energy density; (**c**) Metallographic morphology at 230 J/mm^2^ laser energy density.

Figure 4 illustrates the microstructure of the formed specimens with different laser energy densities. The microstructure is uniformly alpha phase in the shape of a partial flower with fine petals. Figure 4a,b demonstrates that a finer grain is obtained as the laser energy density increases. Figure 4c shows that the molten pool results in a more rounded morphology when the laser energy density reaches 230 J/mm^2^. When the laser power reaches a certain level, the powder materials in the laser action area are heated rapidly, resulting in insufficient energy diffusion, which could easily cause some materials to vaporize directly without the melting stage to produce metal vapor, and the liquid is likely to break up into a row of spheres under the effect of surface tension (“balling” effect [7]). 

**Figure 5:**
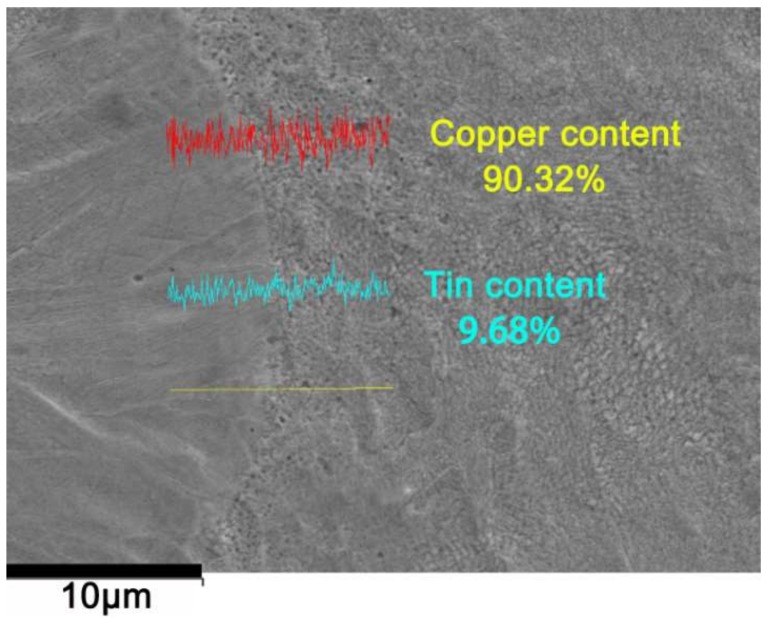
Component analysis at a laser energy density of 220 J/mm^2^.

The composition of the specimen at a laser energy density of 220 J/mm^2^ was analyzed by SEM, as shown in Figure 5. It can be seen that the composition of the microstructure is homogeneous with almost all Sn dissolved into the matrix, and the δ-phase is not formed during the cooling process. Lin et al. [16] applied heat treatment to the tin bronze alloy to dissolve the δ-phase into the α-Cu matrix and thus realized better properties. This research pointed out that the properties of the copper solid solution matrix can be improved as more tin dissolves into the copper solid solution. Considering the plastically deformable property of the alpha copper phase, the plastic properties are significantly strengthened. The maximum growth of yield strength and the elongation are 188% and 217%, respectively, compared with the values of the Chinese Industrial Standards YS/T 545-2006 [15].

On the other hand, the average grain size is 5.1 ± 1.8 μm when the laser energy density is 220 J/mm^2^, which is far lower than that of products made by the traditional casting process, which is around 30 μm [17]. The refined grain size can be ascribed to the higher cooling rate of the sintering process (~10^4^ K/s), compared to that of the traditional casting process (~10^2^ K/s). The growth of the yield strength can be viewed as a Hall-Petch mechanism [18] as well. This states that the deformation of the crystal material depends mainly on the internal dislocation motion and the grain boundary hinders the dislocation motion. Therefore, when the grain size decreases, the hindrance effect on the dislocation motion would be stronger and the proportion of grain boundary would be higher in the material, which leads to a higher yield strength [19].

## 4. Conclusions

Tin bronze bar samples with good mechanical properties were fabricated with CuSn_10_ powder via laser powder bed fusion technology. The density and mechanical properties were found to exhibit a nonlinear relationship with laser energy density, with the best properties achieved at 220 J/mm^2^. “Balling effect” appeared and led to poor properties when the laser energy density was 230 J/mm^2^. The specimen at 220 J/mm^2^ showed good mechanical properties, which were higher than those achieved with the standard continuous casting. The composition was homogeneous with almost all tin dissolved into the copper alpha phase, which accounted for the significantly strengthened plastic properties. The good properties were ascribed to the mechanism of solid solution strengthening and the size effect of the Hall-Petch mechanism.

## Figures and Tables

**Table 1:** Experimental parameters.

Mark	Laser Energy Density/J/mm^2^
A1	190
A2	200
A3	210
A4	220
A5	230

**Table 2:** Mechanical properties at different laser energy densities.

The Standard	Density/g/cm^3^	Hardness/HRB		Yield Strength/MPa		Tensile Strength/Mpa		Elongation %	
YS/T 545-2006	8.6	55		360		170		6	
Laser Energy Density	Measured Value	Measured Value	Increased by	Measured Value	Increased by	Measured Value	Increased by	Measured Value	Increased by
E = 210 J/mm^2^	8.8 ± 0.2	75.5 ± 0.3	37%	369 ± 3	3%	433 ± 3	155%	14 ± 0.6	133%
E = 220 J/mm^2^	8.9 ± 0.1	76.6 ± 0.2	39%	399 ± 2	11%	490 ± 4	188%	19 ± 0.8	217%
E = 230 J/mm^2^	8.7 ± 0.2	74.9 ± 0.2	36%	390 ± 2	8%	486 ± 3	185%	15 ± 0.7	150%

## References

[1] Afshari E., Ghambari M., Abdolmalek H. (2017). Production of CuSn_10_ bronze powder from machining chips using jet milling. Int. J. Adv. Manuf. Technol..

[2] Canakci A., Varol T., Cuvalci H., Erdemir F. (2014). Synthesis of novel CuSn_10_-graphite nanocomposite powders by mechanical alloying. Micro Nano Lett..

[3] Widyastuti, Vicko G.A., Ardhyananta H., Rochman R. (2015). Study of Frangible Cu-Sn Composite by Powder Metallurgy Method. Adv. Mater. Res..

[4] Li T., Yi D., Hu J., Xu J., Liu J., Wang B. (2017). Surface modification of h-BN and its influence on the mechanical properties of CuSn 10/h-BN composites. J. Alloys Compd..

[5] Dong X.J., Wang L.M., Zhang J.H., Liu Y.H., Wang L.S. (2010). Influence of morphology of different partially alloyed CuSn10 powders on the sintering character self-lubricated bearings. Powder Metall..

[6] Hui L.I., Wang L., Wan X., Zhan L.I., Bai (2003). Study on preparation of partially alloyed CuSn10 powders by diffusion treatment. Powder Metall. Ind..

[7] Simchi A., Petzoldt F., Pohl H. (2003). On the development of direct metal laser sintering for rapid tooling. J. Mater. Process. Technol..

[8] Wang Y., Shen Y., Wang Z., Yang J., Liu N., Huang W. (2010). Development of highly porous titanium scaffolds by selective laser melting. Mater. Lett..

[9] Attar H., Prashanth K.G., Chaubey A.K., Calin M., Zhang L.C., Scudino S., Eckert J.L. (2015). Comparison of wear properties of commercially pure titanium prepared by selective laser melting and casting processes. Mater. Lett..

[10] Baraton M.I., Merhari L., Ferkel H., Castagnet J.F. (2002). Comparison of the gas sensing properties of tin, indium and tungsten oxides nanopowders: Carbon monoxide and oxygen detection. Mater. Sci. Eng. C.

[11] Liu Z.H., Zhang D.Q., Sing S.L., Chua C.K., Loh L.E. (2014). Interfacial characterization of SLM parts in multi-material processing: Metallurgical diffusion between 316L stainless steel and C18400 copper alloy. Mater. Charact..

[12] Wang L., Wang S., Wu J. (2017). Experimental investigation on densification behavior and surface roughness of AlSi10Mg powders produced by selective laser melting. Opt. Laser Technol..

[13] Mao Z., Zhang D.Z., Wei P., Zhang K. (2017). Manufacturing Feasibility and Forming Properties of Cu-4Sn in Selective Laser Melting. Materials.

[14] Zhang H., Zhu H., Qi T., Hu Z., Zeng X. (2016). Selective laser melting of high strength Al–Cu–Mg alloys: Processing, microstructure and mechanical properties. Mater. Sci. Eng. A.

[15] National Technical Committee for Standardization of Nonferrous Metals (2008). Standard Collection of Copper and Copper Alloy: Method Volume 2008.

[16] Lin G.B., Wang Z.D., Zhang W., Li W.Q., Zhang H. (2011). Effects of heat treatment on microstructure and performance of tin bronze alloy. Foundry.

[17] Li Y.Z. (1993). Rapid Solidification Technology and Materials.

[18] Scudino S., Unterdörfer C., Prashanth K.G., Attar H., Ellendt N., Uhlenwinkel V. (2015). Additive manufacturing of Cu–10Sn bronze. Mater. Lett..

[19] Heo N.H., Heo Y.U., Kwon S.K., Kim N.J., Kim S.J., Lee H.C. (2018). Extended Hall–Petch Relationships for Yield, Cleavage and Intergranular Fracture Strengths of bcc Steel and Its Deformation and Fracture Behaviors. Met. Mater. Int..

